# Supplementary material for: Identification and Characterization of a Novel N- and O-Glycosyltransferase from Saccharopolyspora erythraea
Source: Molecules. 2020 Jul 27;25(15):3400. doi: 10.3390/molecules25153400 (PMC7435583; doi:10.3390/molecules25153400)
Supplement: Supplementary file 1 [file molecules-25-03400-s001.pdf]

molecules

Supplementary Material

Supplementary File 1: Construction of plasmids

Note: Restriction sites within the primers are marked in bold face.

1. pKCΔ*sace_3599* for the inactivation of *sace_3599* in the genome of *S. erythraea*

S. erythraea genomic DNA was used as template to amplify homologous regions upstream and downstream of *sace_3599*. The upstream region was amplified using the primers sace_3599-NX-f, 5′–TATA**G CGGCC GC**GTG GCCCA CCTCA AGCAG, and sace_3599-NX-r, 5′–GCGC**T CTAGA** GTCGG TCATG GGTGG GACGT C, and cloned into the NotI/XbaI digested vector pKC1132. The downstream region was generated using the primers sace_3599-XP-f, 5′–GCGC**T CTAGA** GCACC GCGTT CAACA AGC, and sace_3599-XP-r, 5′–TATA**C TGCAG** CGTGT CGGAG CGGCG GACC, and the XbaI/PstI digested downstream region was cloned into the plasmid above. The spectinomycin resistance cassette was amplified using the plasmid pLERE-spec-oriT as template and the primers spec-f, 5′–TCGCC CATG**G CATGC T**TGGT CACCA CCGAC TATTT G, and Spec-r, 5′–CGGCC TCGAG **TCTAG A**ACAA TTGCT TATTT GCCGA CTACC. After XbaI/SphI digestion the spectinomycin resistance-conferring gene was cloned into the XbaI/SphI digested plasmid between the two homologous regions.

2. pTOS(z)-*sace_3599* for the complementation of *S. erythraea* Δ*sace_3599* with an intact copy of *sace_3599*

Genomic DNA of *S. erythraea* was used as template for the amplification of *sace_3599*. The intact copy of *sace_3599* was generated using the primers 3599c-f, 5′–GCGC**A CTAGT** AACAC ATGTT CAACA GCGTC GG, and 3599c-r, 5′–GCTA**G AATTC** CAGGA GGAAC ACGAC CATGA CCC. After SpeI/EcoRI restriction *sace_3599* was cloned into the SpeI/EcoRI digested plasmid pTOS(z).

3. Heterologous test system *S. albus* Gluc

3.1. pTOS-Gluc

The gene *oleS* was amplified using pTOS-Rham as template and the primers oleS-f, 5′–TAT**AT GCAT**G ACCCG GCATC CTGCC GGACT AC, and oleS-r, 5′–TGCA**G AATTC** CAGCA GGTGG TGCAC GTAG. The dTDP-L-rhamnose biosynthetic genes *oleS*, *oleE*, *oleL*, and *oleU* were cut out of pTOS-Rham plasmid using NsiI/EcoRI. After NsiI/EcoRI restriction *oleS* was cloned into the NsiI/EcoRI digested plasmid. The resulting plasmid pTOS-Gluc contains *oleS* under control of the *ermE** promoter.

3.2. pUWL-A-sace_3599

The glycosyltransferase gene *sace_3599* of *S. erythraea* was amplified from genomic DNA using the primers sace_3599-f, 5′–TATA**A AGCTT** GCACA CCCTC GCGAT CACC, and sace_3599-r, 5′–GATA**G GATCC** CACGA CCATG ACCCA GAAC, and cloned via HindIII/BamHI digestion into pUWL-A, resulting in the plasmid pUWL-A-*sace_3599*.

4. pET28a-sace_3599-N-his$_6$ for protein synthesis of Sace_3599

The glycosyltransferase gene *sace_3599* was amplified using genomic DNA of *S. erythraea* as template and the primers pET-sace_3599-f, 5′–TACG**C ATATG** ACCAA GCACT TCGCG TTCGT CTCCC, and pET28-sace_3599-r, 5′–TAGC**A AGCTT** TCAGG CCAGG TAGGA CTCCA G. The PCR product was blunt end subcloned into EcoRV digested pBSK$^-$ vector. After NdeI/HindIII digestion *sace_3599* was cloned into the NdeI/HindIII digested vector pET28a(+).

Supplementary File 2: NMR data for structure elucidation of U3G

Table S1: NMR data of **U3G**[1] (600/150 MHz (DMSO-d_6. 35 °C)

Pos.	δ_C	δ_H (*J* Hz) [ppm]	COSY	^{1}H,^{13}C-HMBC
1	144.9			3-H, 1′-H
1-NH	-	10.82 d (6.4)		
2	125.1	7.42 d (9.6)	3-H	1-NH, ^{1}J, 3-H
3	129.0	7.30 d (9.6)	2-H	2-H, ^{1}J
4	146.7			2-H
4a	108.0			3-H
5	125.9	8.25 m	6-H	6-H, 7-H
6	132.7	7.82 m	5-H	5-H, 7-H, 8-H
7	132.6	7.81 m	8-H	5-H, 6-H, 8-H
8	125.7	8.24 m	7-H	6-H, 7-H
8a	133.8			5-H, 7-H, 8-H
9	181.5			(2-H), 8-H
9a	109.4			1-NH, 2-H
10	182.3			(3-H), 5-H
10a	133.9			5-H, 6-H, 8-H
1′	83.2	4.72 d (8.4)	2′-H	1-NH, 2′-H
2′	73.6	3.23 dd (8.8, 8.4)	1′-H, 3′-H	1-NH, 1′-H, 3′-H
3′	77.6	3.32 dd (9.0, 8.8)	2′-H, 4′-H	1′-H, 2′-H
4′	70.1	3.20 dd (9.2, 9.0)	3′-H, 5′-H	3′-H, 6′-H_a, 6′-H_b
5′	77.9	3.36 ddd (9.2, 6.0,	4′-H, 6′-H_a, 6′-	4′-H, 6′-H_b
6′	60.9	H_a: 3.69 dd (12.0,	5′-H, 6′-H_b	4′-H, 5′-H

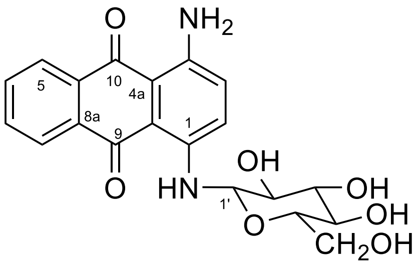

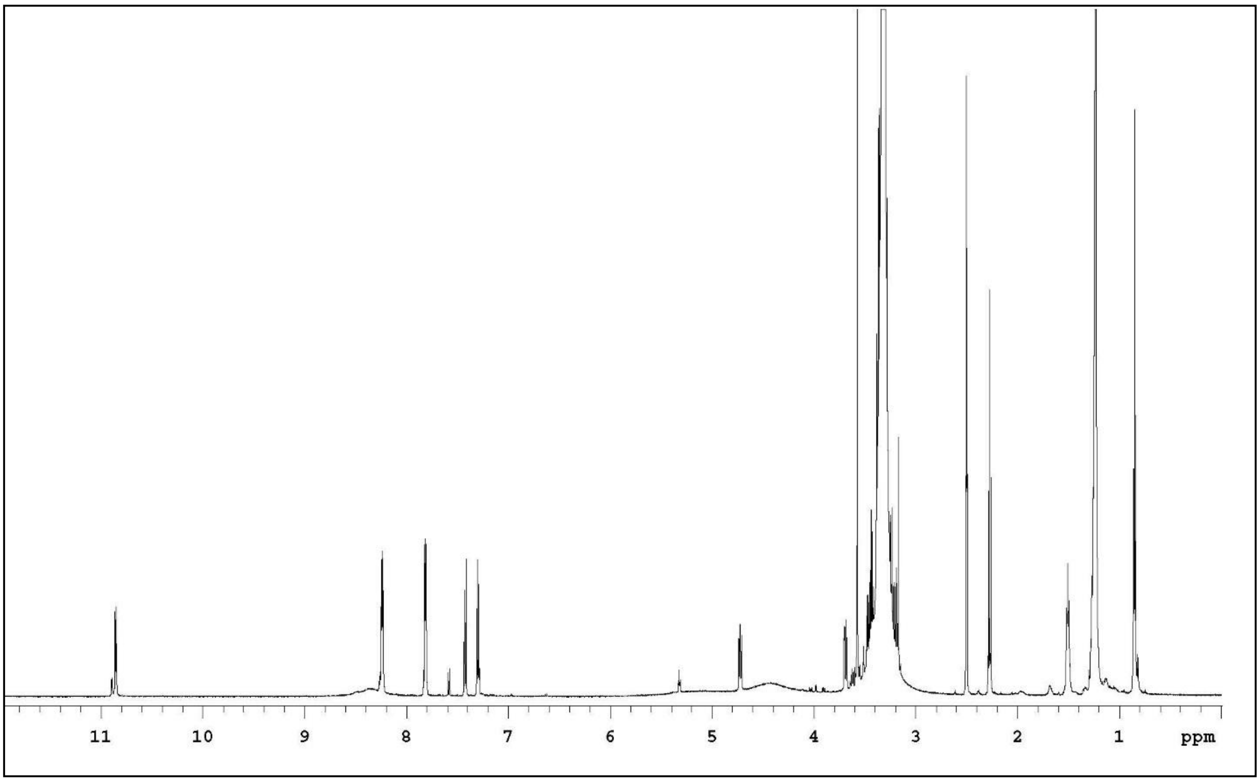

Figure S1: ^{1}H NMR spectrum of **U3G**[1] (600 MHz, DMSO-d_6, 35 °C).

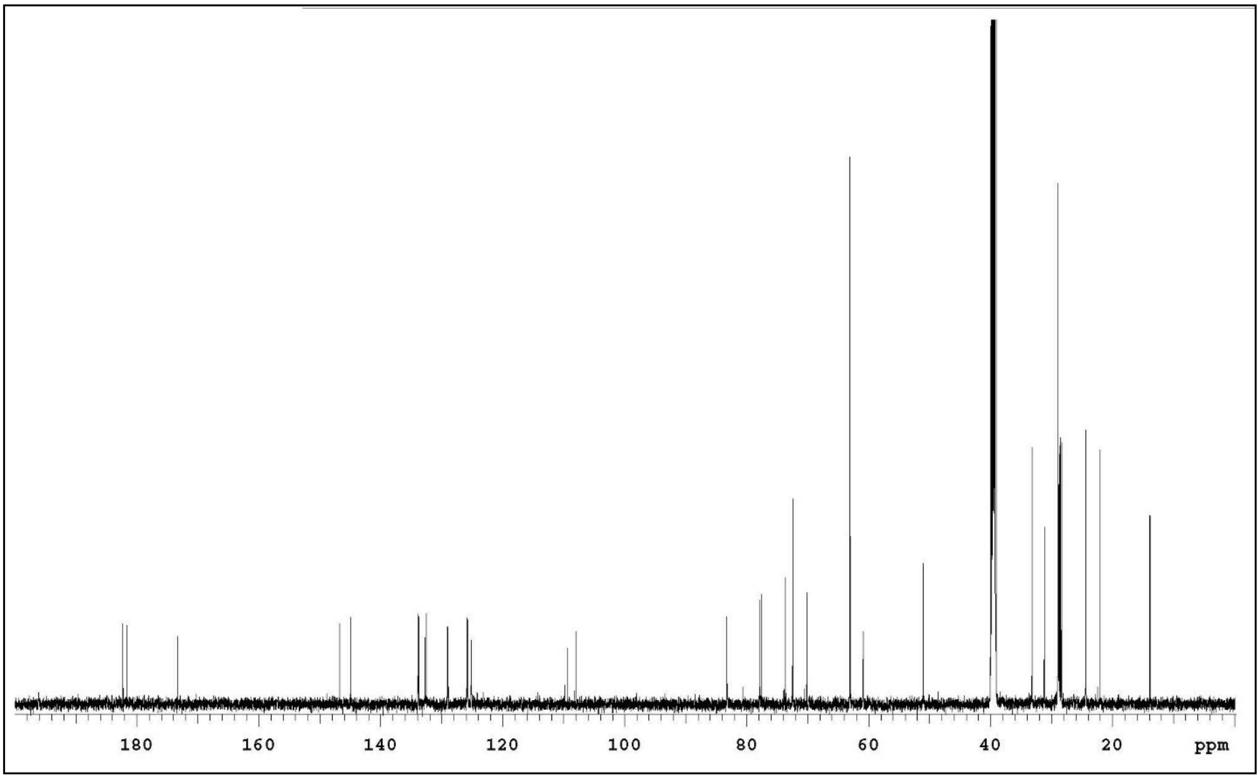

Figure S2: ^{13}C NMR spectrum of **U3G**[1] (150 MHz, DMSO-d_6, 35 °C).

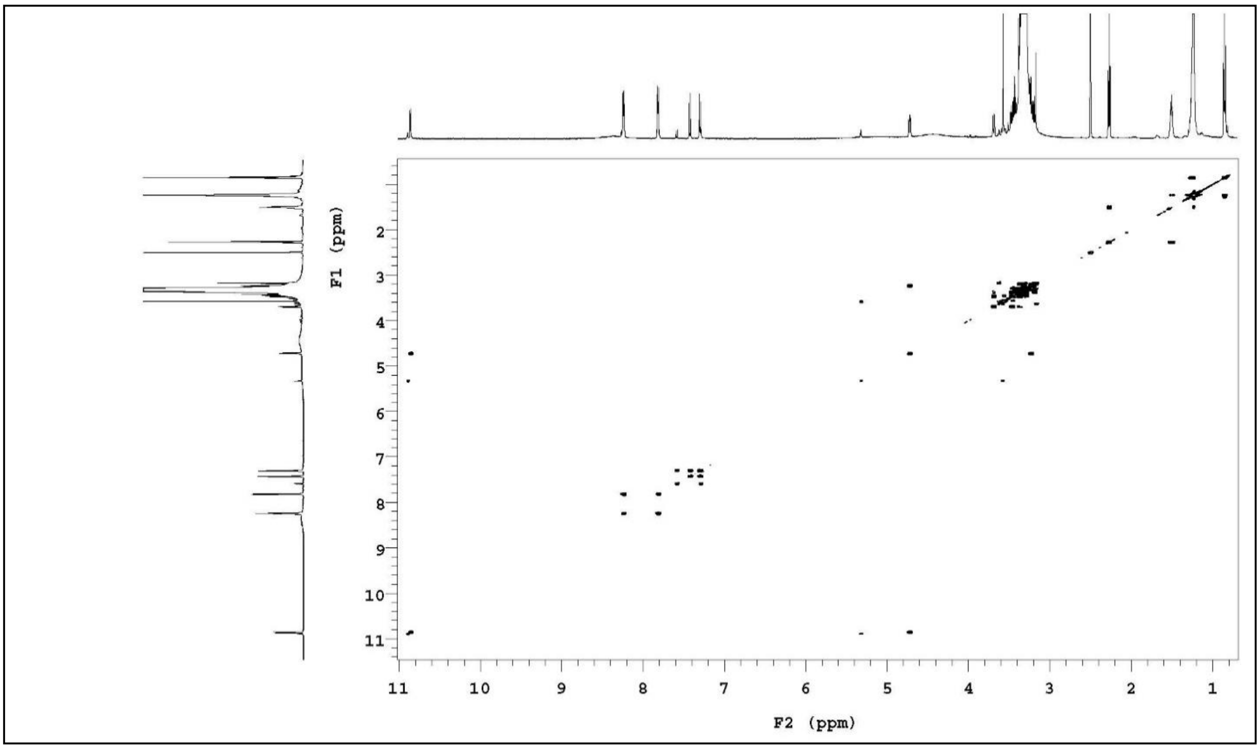

Figure S3: COSY NMR spectrum of **U3G[1]** (600 MHz, DMSO-d$_6$, 35 °C).

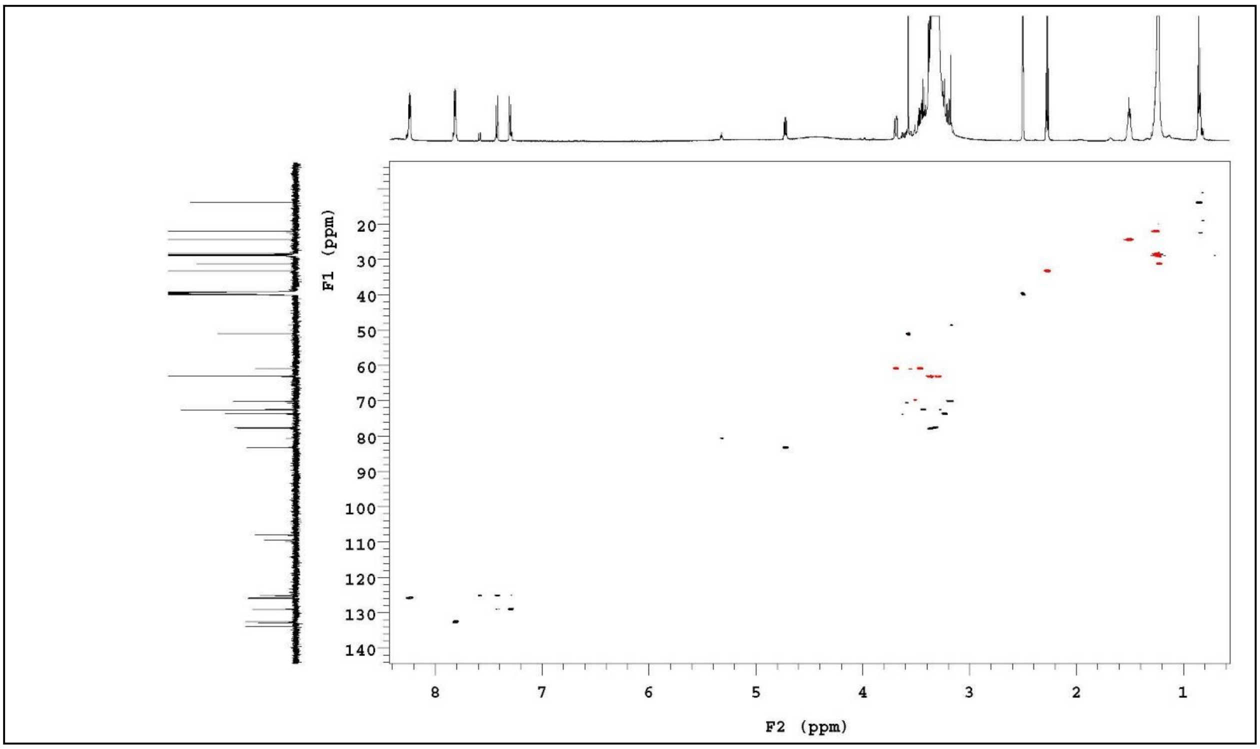

Figure S4: HSQC NMR spectrum of **U3G[1]** (600 MHz, DMSO-d$_6$, 35 °C).

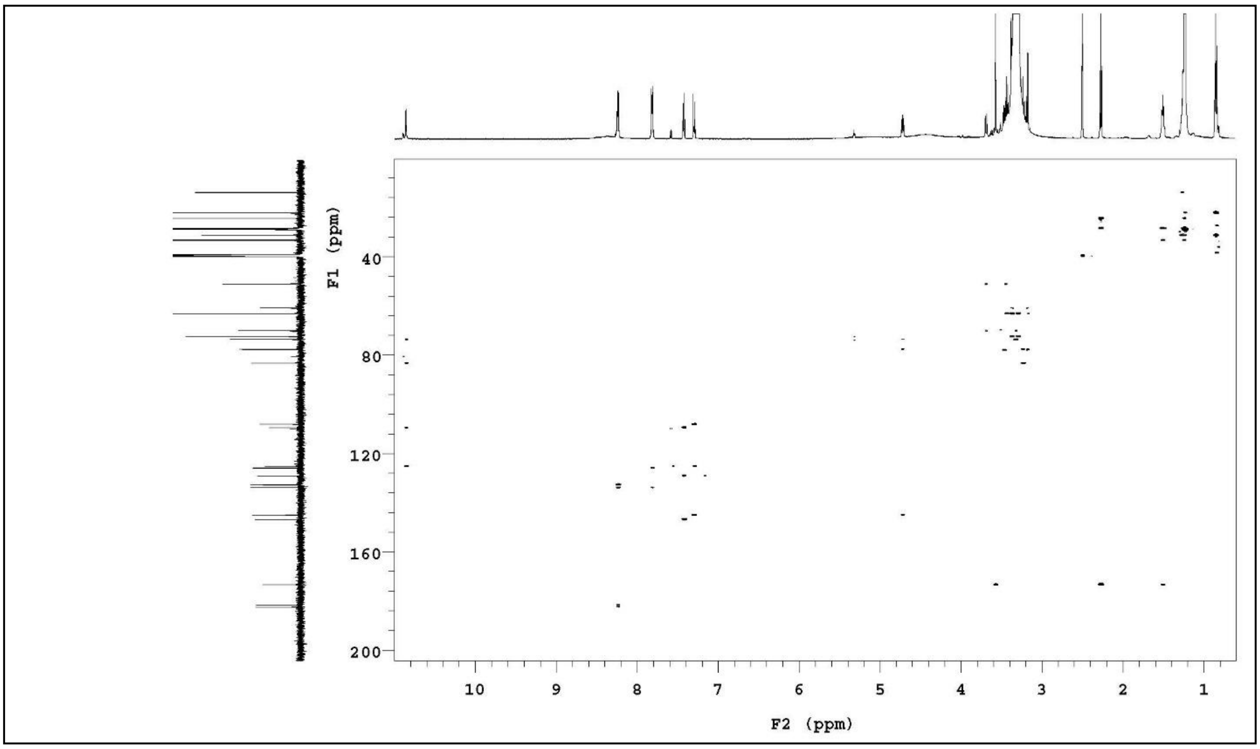

Figure S5: HMBC NMR spectrum of **U3G**[1] (600 MHz, DMSO-d_6, 35 °C).

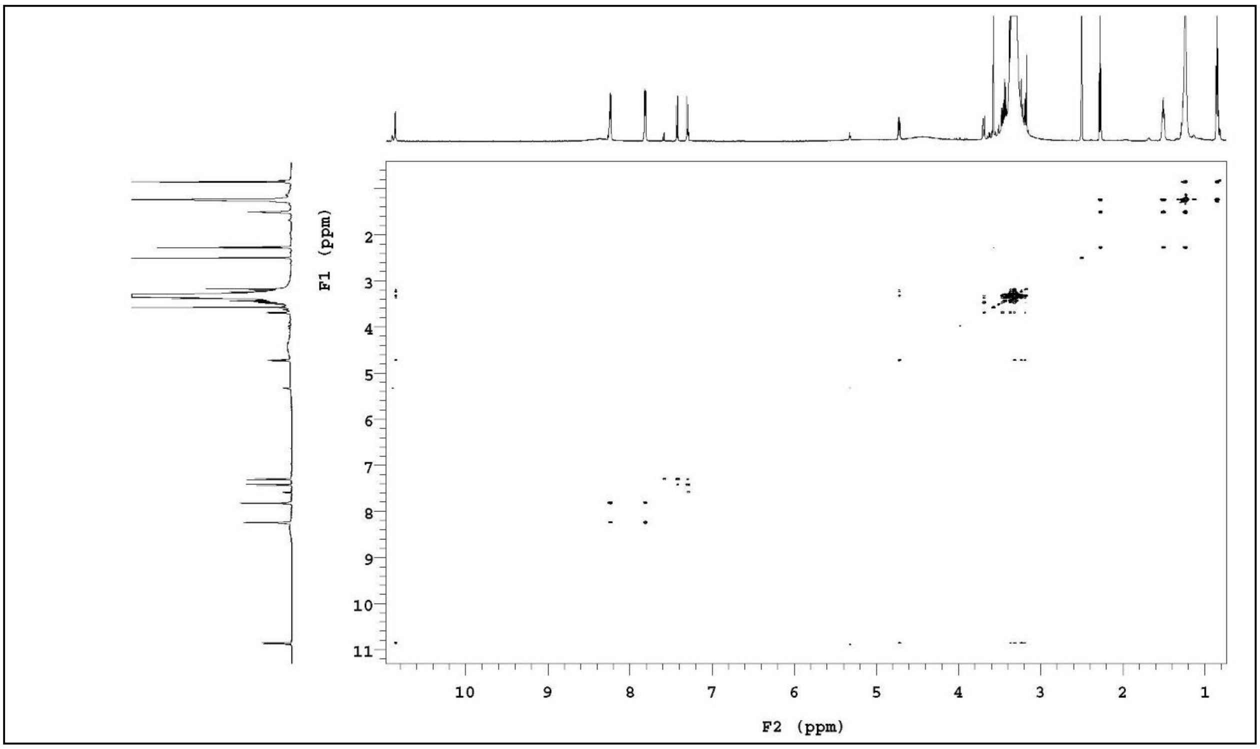

Figure S6: TOCSY NMR spectrum of **U3G**[1] (600 MHz, DMSO-d_6, 35 °C).

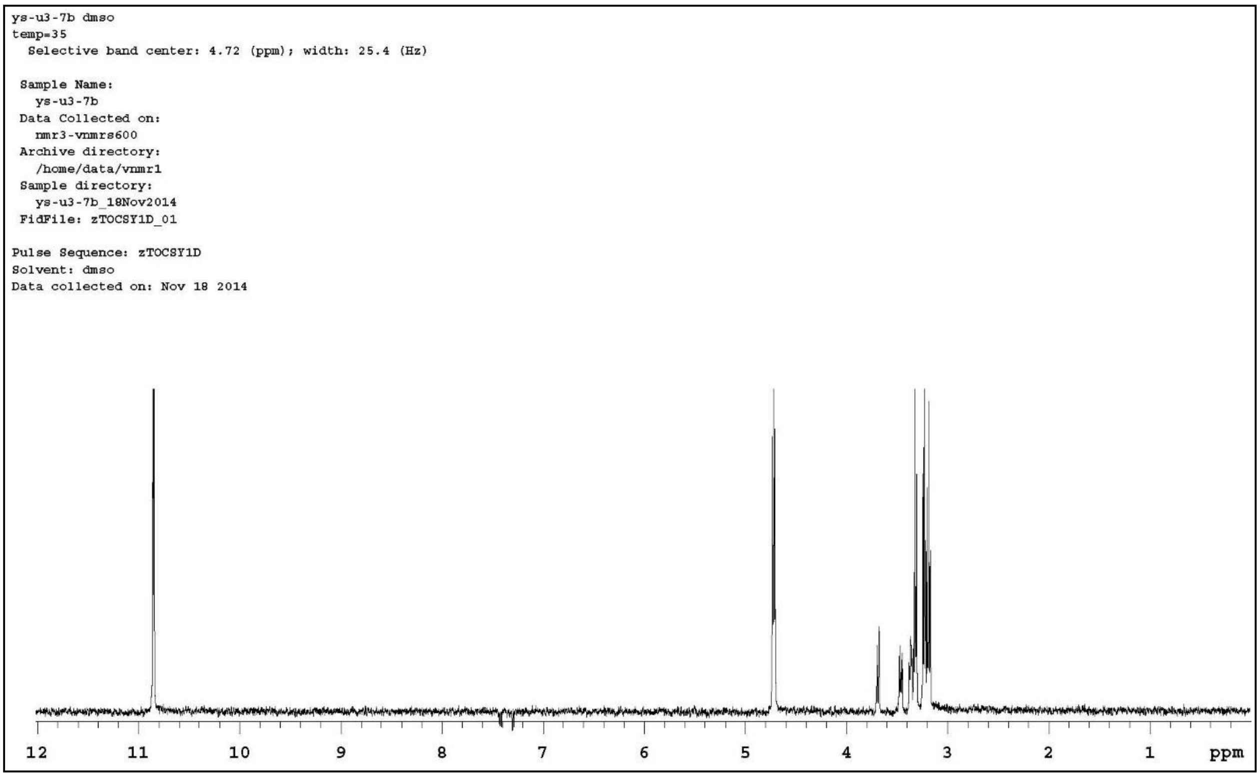

Figure S7: 1D TOCSY NMR spectrum of **U3G¹** (600 MHz, DMSO-d₆, 35 °C, sel. excitation of 1′-H).

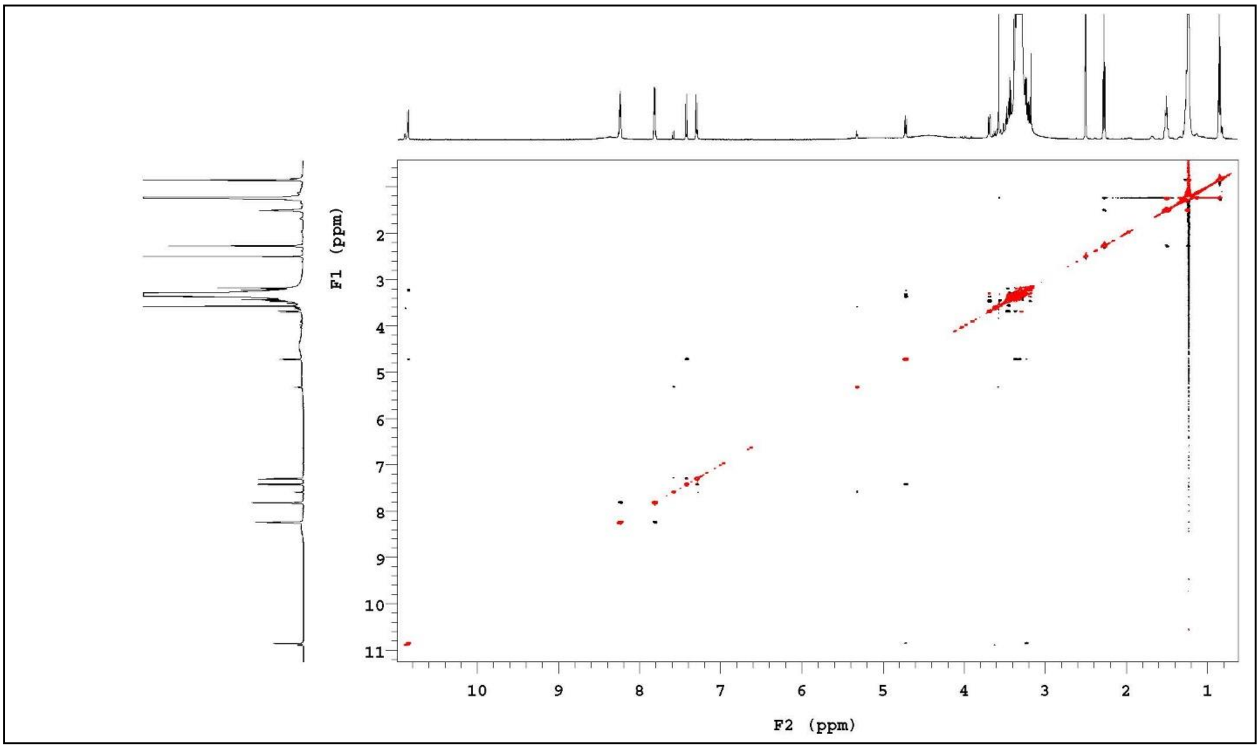

Figure S8: ROESY NMR spectrum of **U3G¹** (600 MHz, DMSO-d₆, 35 °C, sel. excitation of 1′-H).

Table S2: NMR data of **U3G^2** (600/150 MHz (DMSO-d_6. 35 °C)

Pos.	δ_C	δ_H (*J* Hz) [ppm]	COSY	^{1}H,^{13}C-HMBC
1	144.9			2-H, 1′-H
1-NH	-	10.89 d (4.8)	1′-H	
2	125.2	7.59 d (9.7)	3-H	1-NH
3	129.0	7.29 d (9.7)	2-H	
4	146.7			2-H
4a	108.2			3-H
5	125.9	8.24 m	6-H	6-H, 7-H
6	133.8	7.82 m	5-H, 7-H	5-H, 7-H
7	133.6	7.82 m	6-H, 8-H	6-H, 8-H
8	125.9	8.24 m	7-H	6-H, 7-H
8a	133.8			7-H, 8-H
9	181.7			8-H
9a	109.8			1-NH, 2-H
10	182.3			5-H
10a	133.8			5-H, 6-H
1′	80.7	5.33 t (4.8)*	1-NH, 2′-H	1-NH
2′	70.3	3.59 dd (8.9, 4.8)*	1′-H, 3′-H	(1-NH), 1′-H
3′	73.7	3.63 dd (9.4, 8.9)*	2′-H, 4′-H	1′-H, 2′-H, 4′-H
4′	70.2	3.17 dd (9.5, 8.9)*	3′-H, 5′-H	3′-H
5′	72.2	3.29 m**	4′-H, 6′-H_b	1′-H, ^1J, 4′-H
6′	61.0	H_a: 3.56 m	6′-H_b	4′-H

*coupling constants in the sugar system are taken from ^{1}H NMR spectrum and 1D-TOCSY on 5.33 ppm

**covered from water

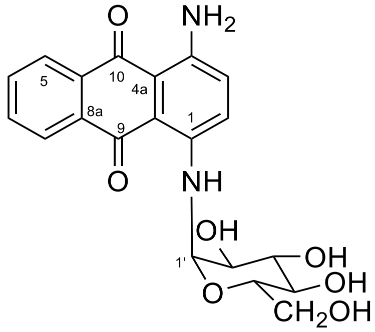

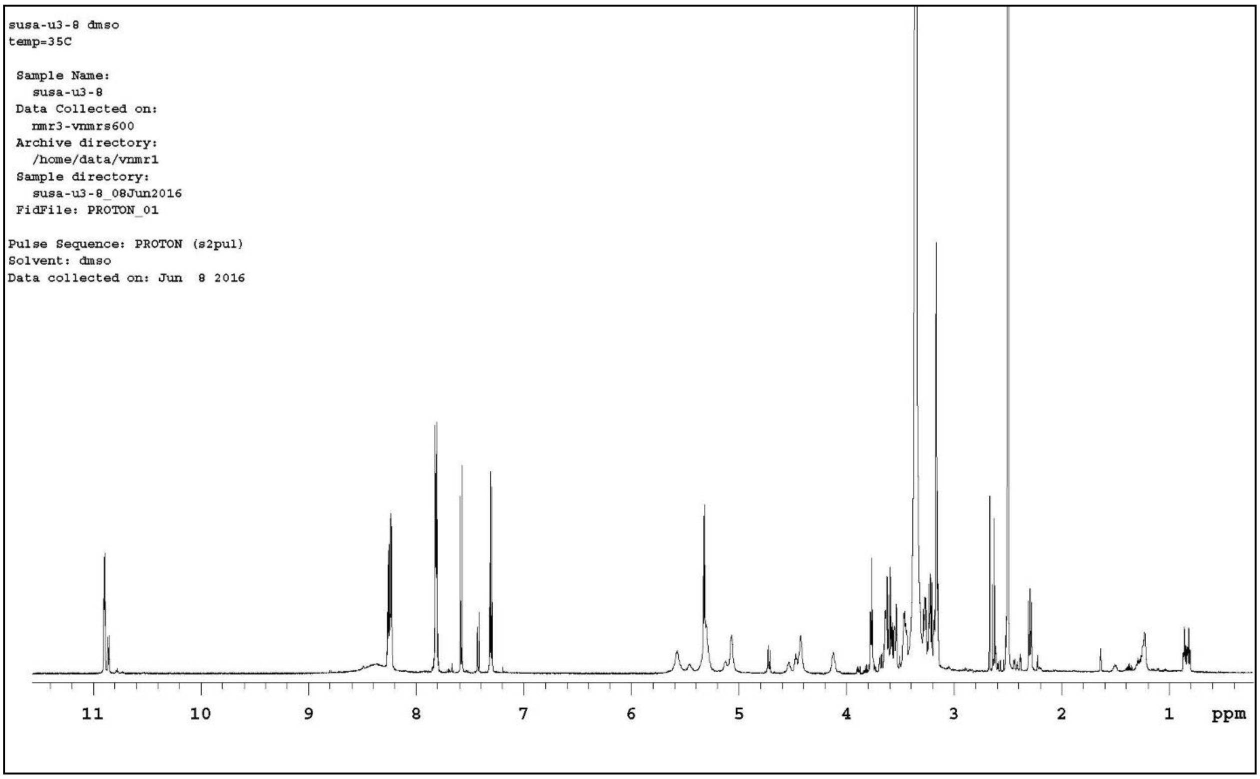

Figure S9: ^{1}H NMR spectrum of **U3G²** (600 MHz, DMSO-d$_6$, 35 °C).

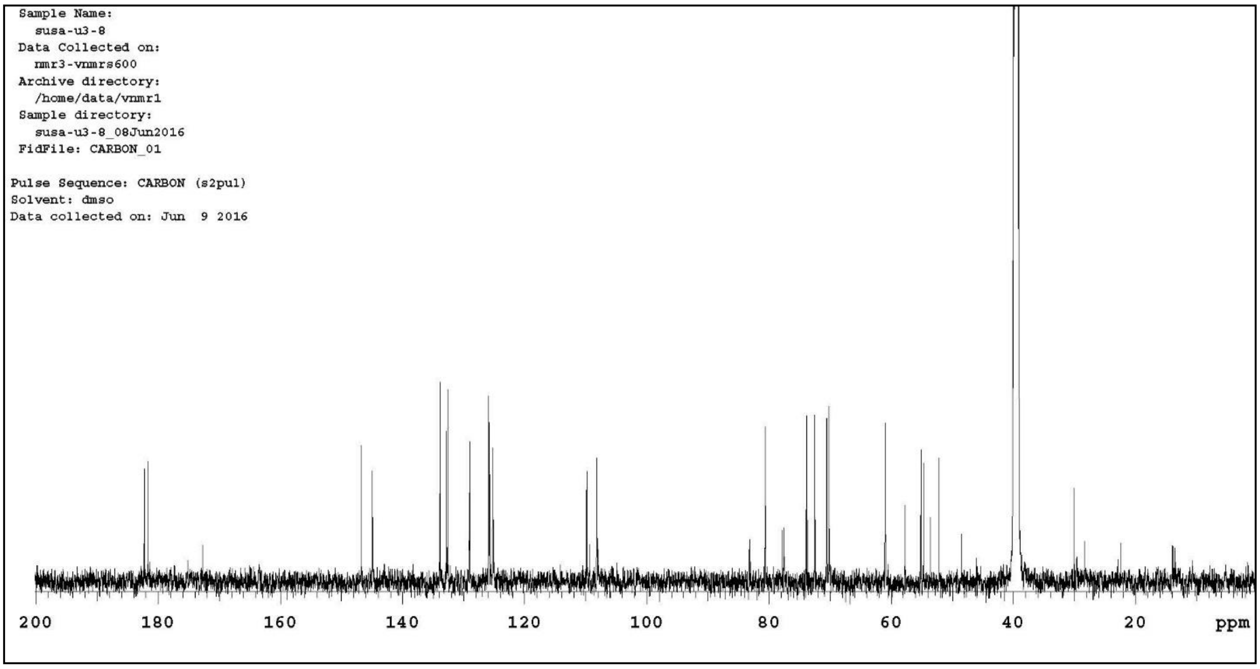

Figure S10: ^{13}C NMR spectrum of **U3G²** (150 MHz, DMSO-d$_6$, 35 °C).

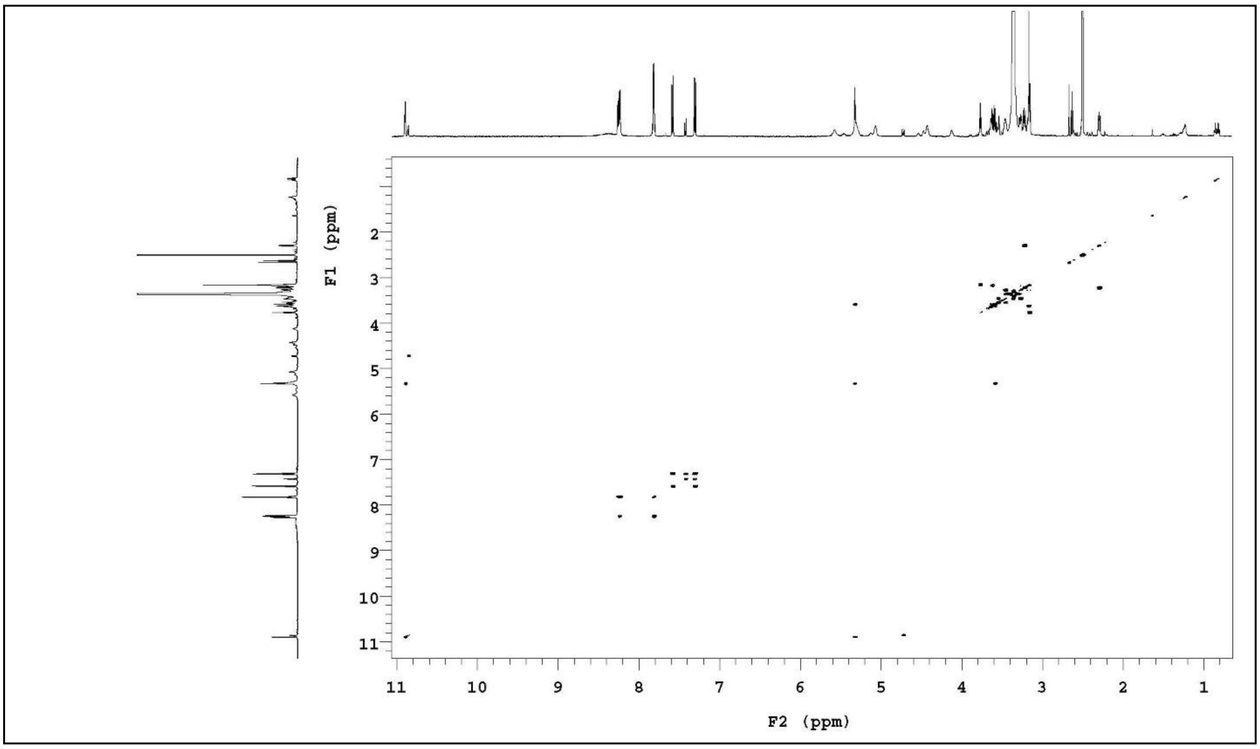

Figure S11: COSY NMR spectrum of **U3G²** (600 MHz, DMSO-d₆, 35 °C).

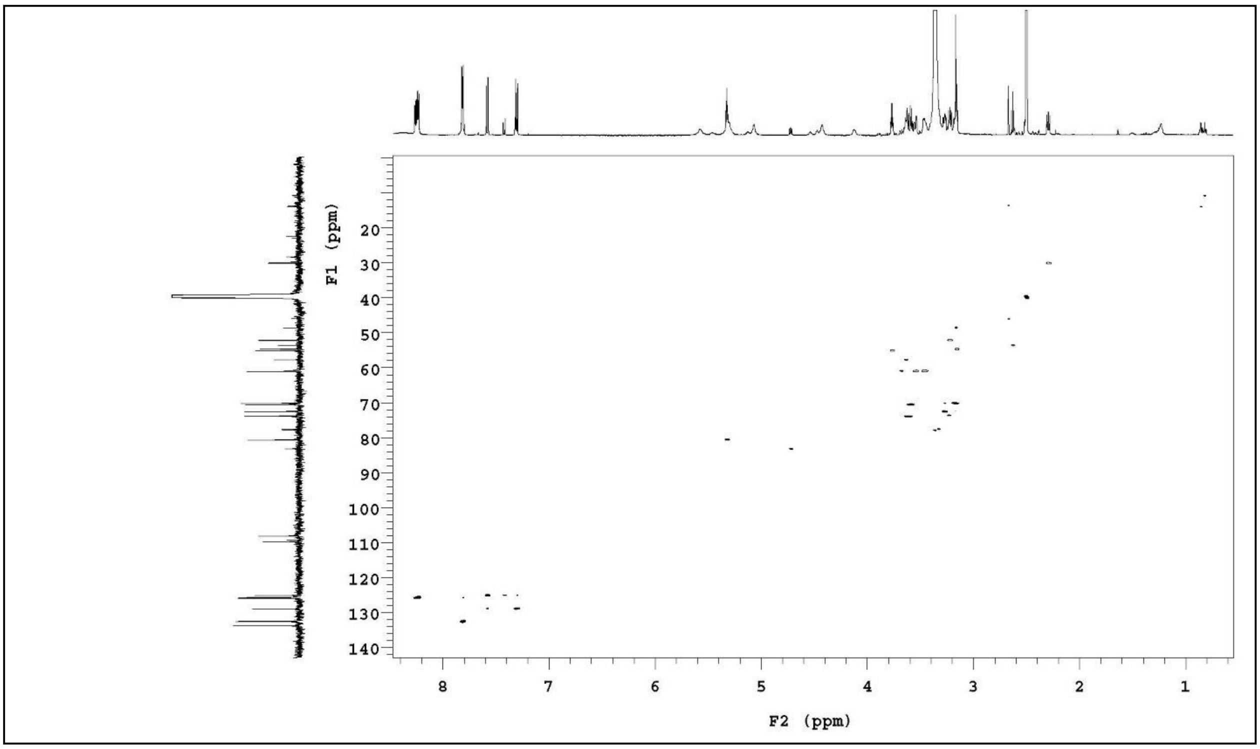

Figure S12: HSQC NMR spectrum of **U3G²** (600 MHz, DMSO-d₆, 35 °C).

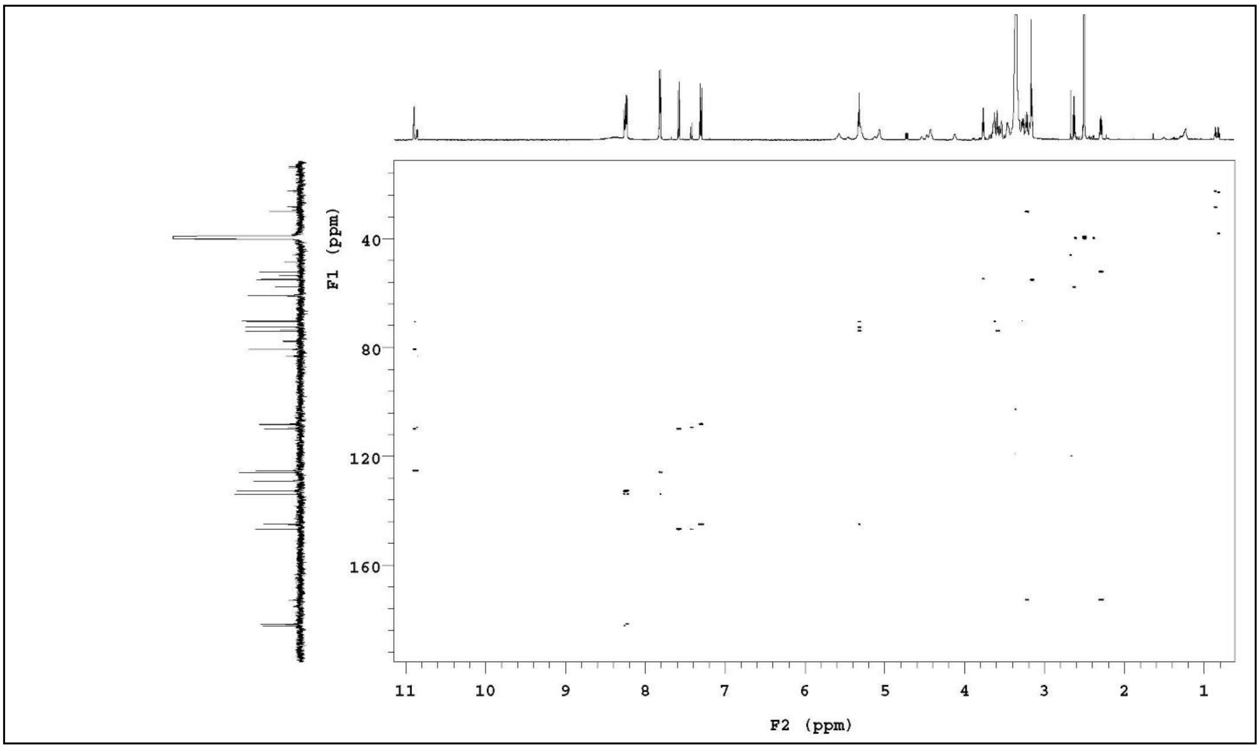

Figure S13: HMBC NMR spectrum of **U3G²** (600 MHz, DMSO-d₆, 35 °C).

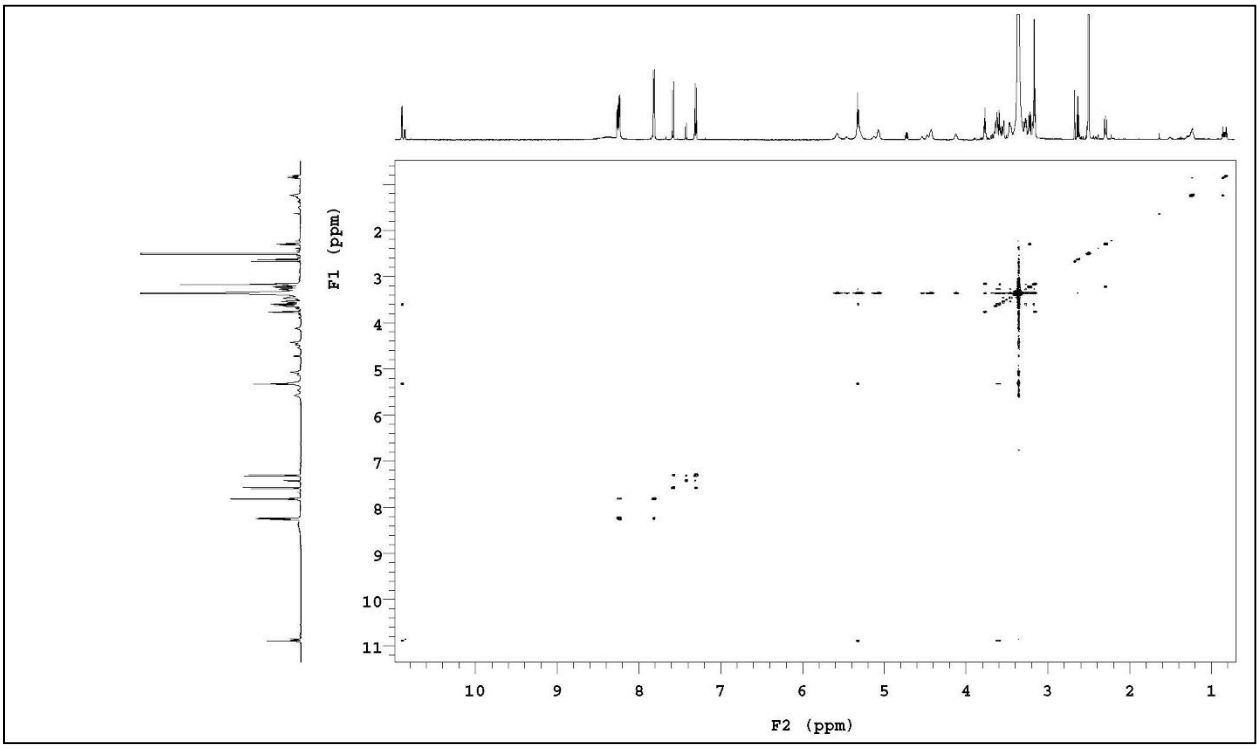

Figure S14: TOCSY NMR spectrum of **U3G²** (600 MHz, DMSO-d₆, 35 °C).

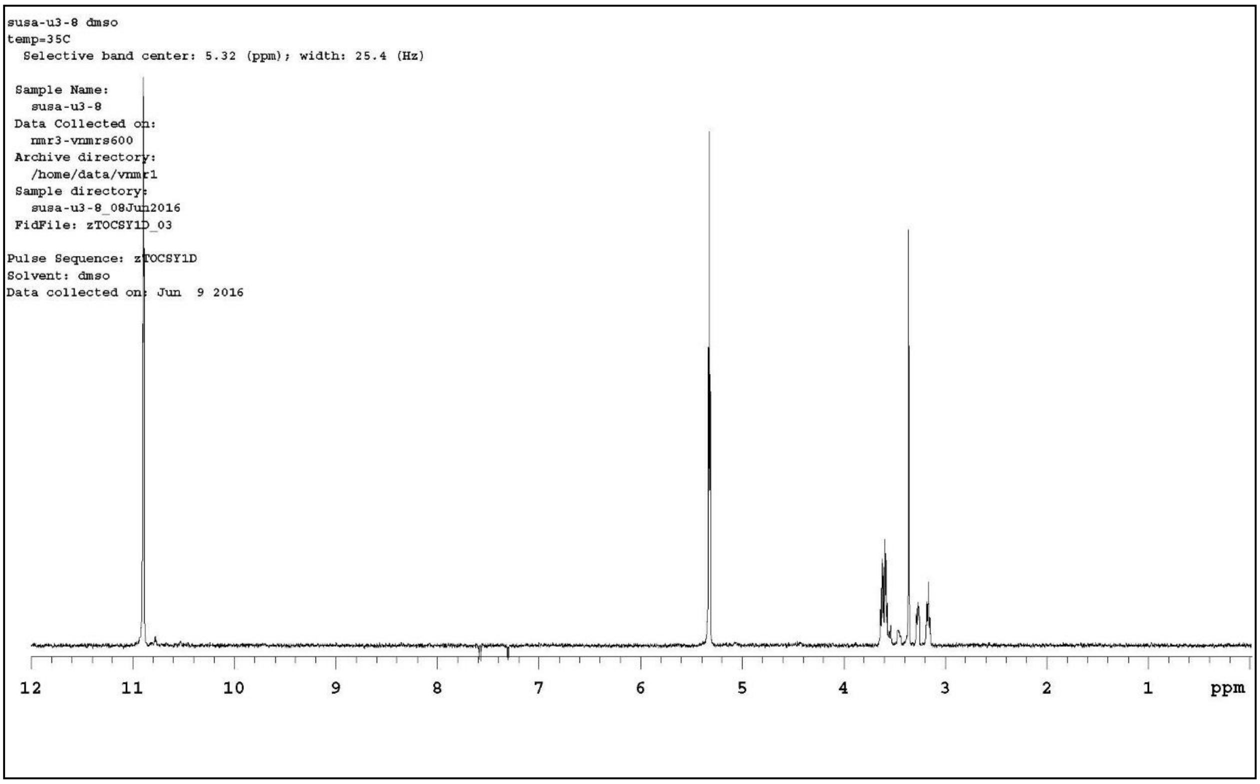

Figure S15: 1D TOCSY NMR spectrum of **U3G²** (600 MHz, DMSO-d$_6$, 35 °C, sel. excitation of 1′-H).

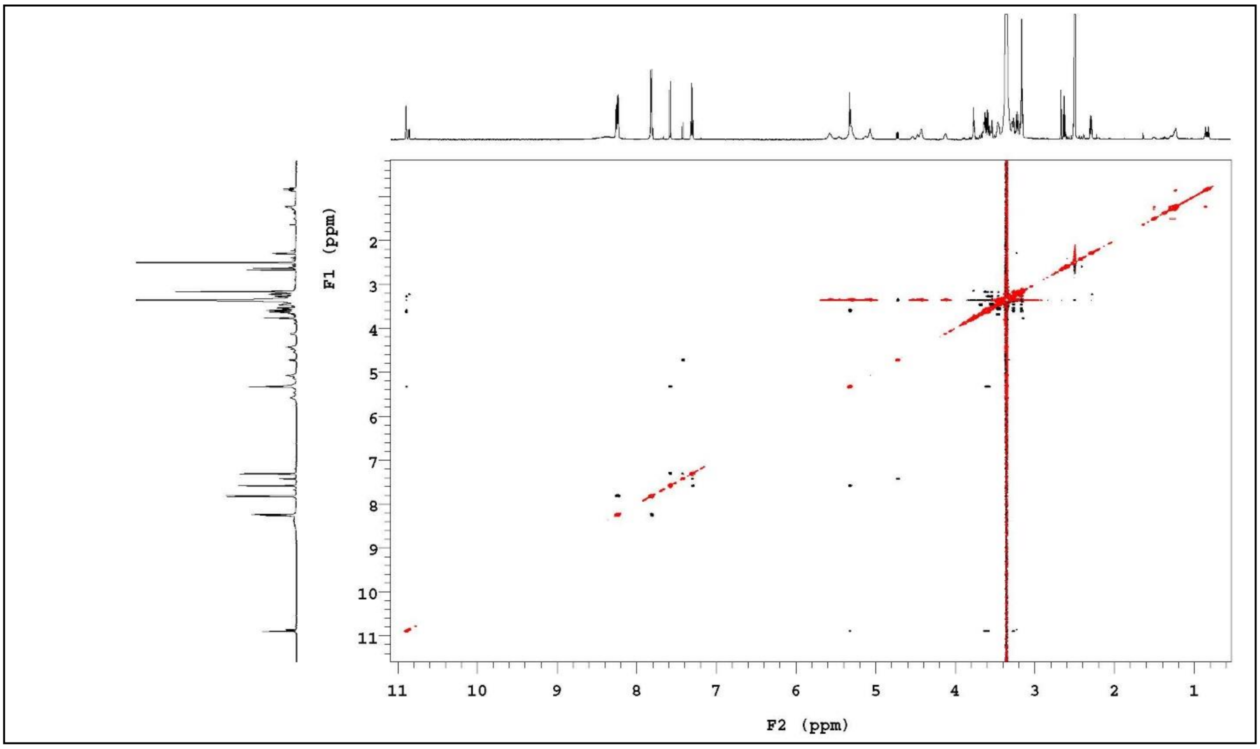

Figure S16: ROESY NMR spectrum of **U3G²** (600 MHz, DMSO-d$_6$, 35 °C).

Supplementary File 3: Protein purification of Sace_3599

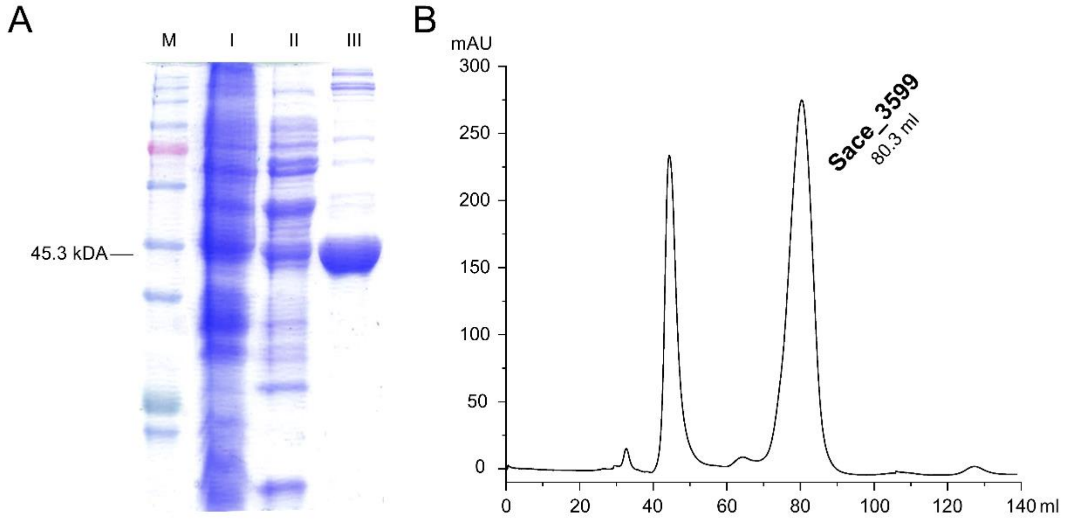

Figure S17: Protein purification

(A) SDS-PAGE gel showing fractions of the Ni^{2+}-NTA affinity chromatography of Sace_3599. Line I belongs to the flow through, line II to the washing with 15 % elution buffer B, line III to the elution of the protein Sace_3599 with 50 % elution buffer B. Line M shows the Protein marker VI (10-245) of NEB. (B) Chromatogram of the gel filtration of Sace_3599 (280 nm). Sace_3599 is eluting at 72.5–90 ml.

Supplementary File 4: Factors affecting the biotransformation activity of Sace_3599

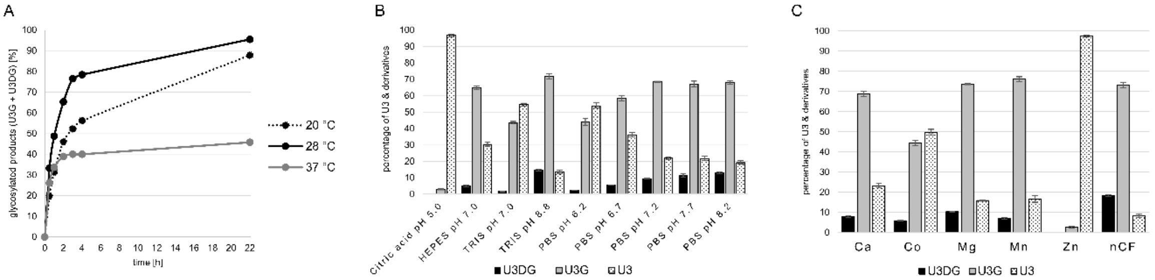

Figure S18: Factors affecting the biotransformation activity
(**A**) Temperature and time dependence of biotransformation activity of Sace_3599 in *in vitro* activity assays. (**B**) Dependence on the buffer and the pH value of the in vitro biotransformation activity of Sace_3599. Standard deviations of three independent experiments are indicated. (**C**) Effect of divalent metal ions on the in vitro biotransformation activity of Sace_3599 pretreated with 10 mM EDTA for 1 h. Standard deviations of three independent experiments are indicated. nCF, no cofactor.

Supplementary File 5: NMR-data eliminating mutarotation/epimerization in U3G

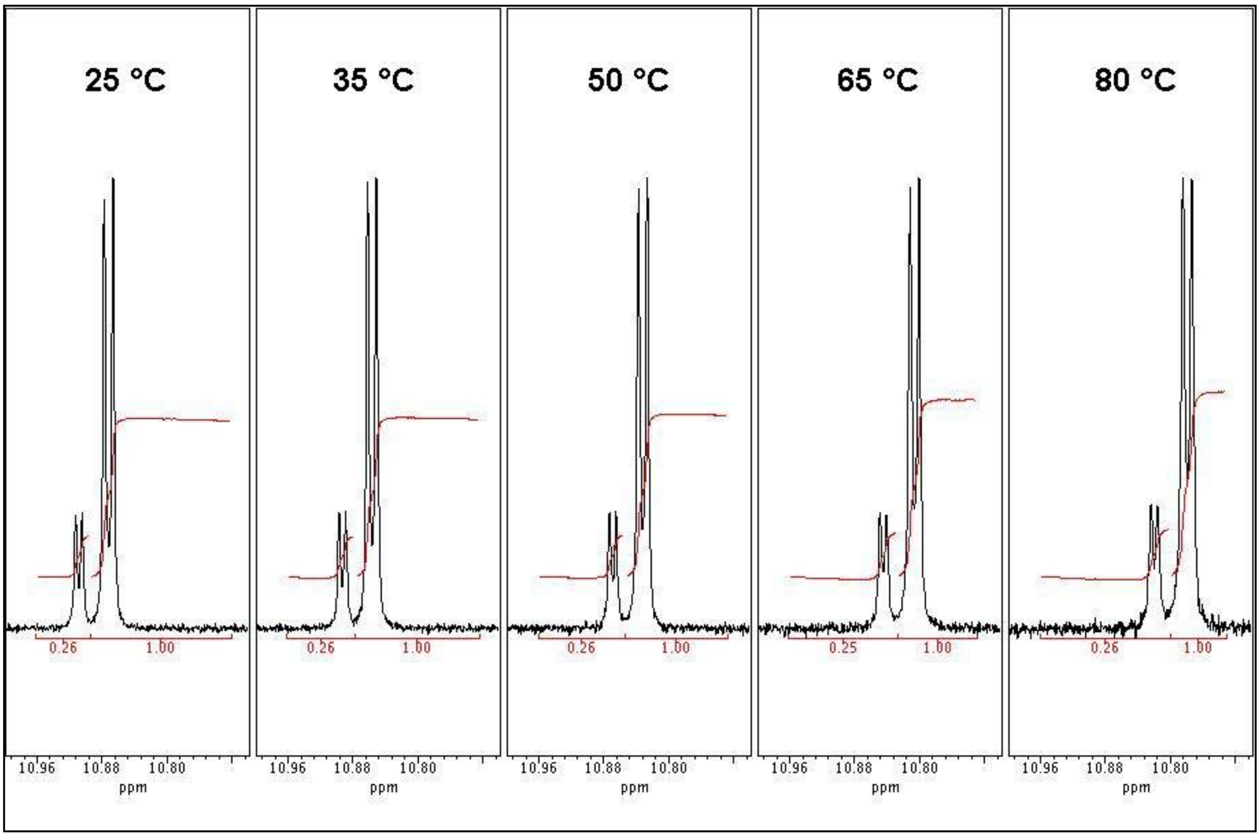

Figure S19: Expansion of NH region of ^{1}H spectra NMR of **U3G¹** (600MHz, DMSO-d_6) at different temperatures.

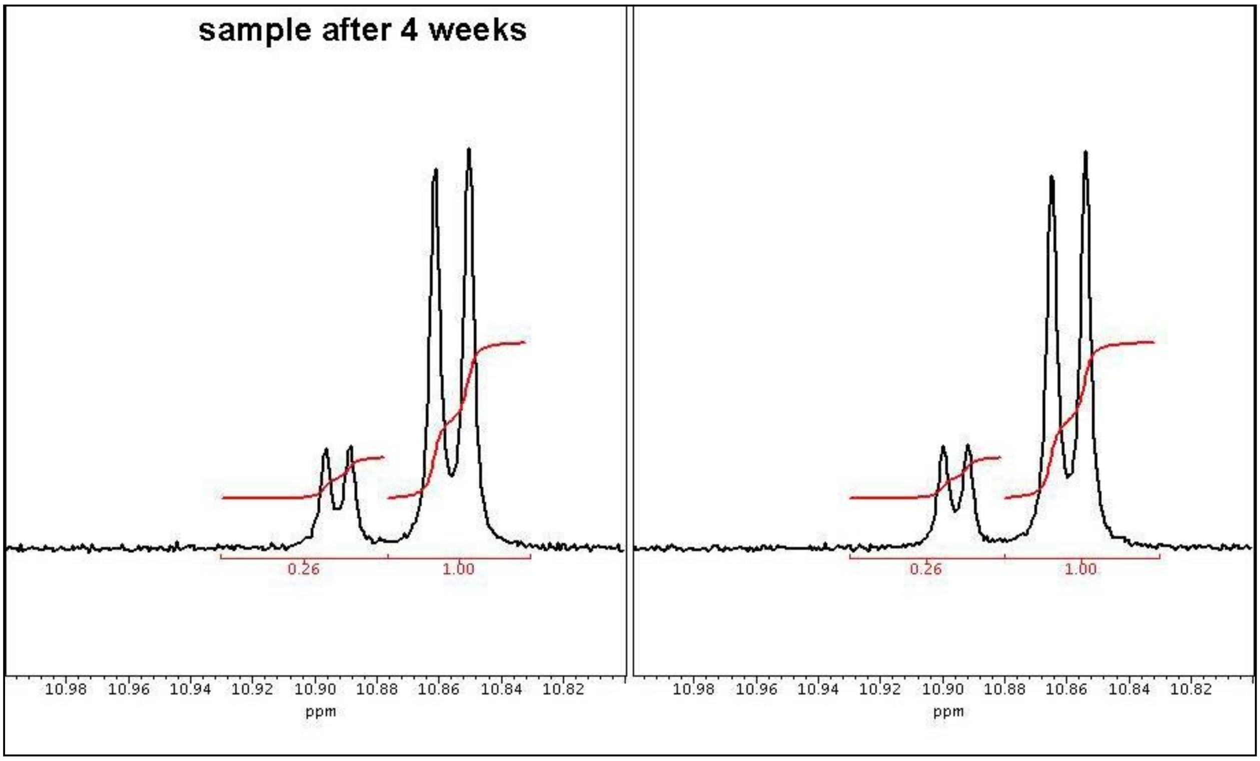

Figure S20: Expansion of NH region of ^{1}H spectra NMR of **U3G¹** after sample preparation and 4 weeks later (600MHz, DMSO-d_6).

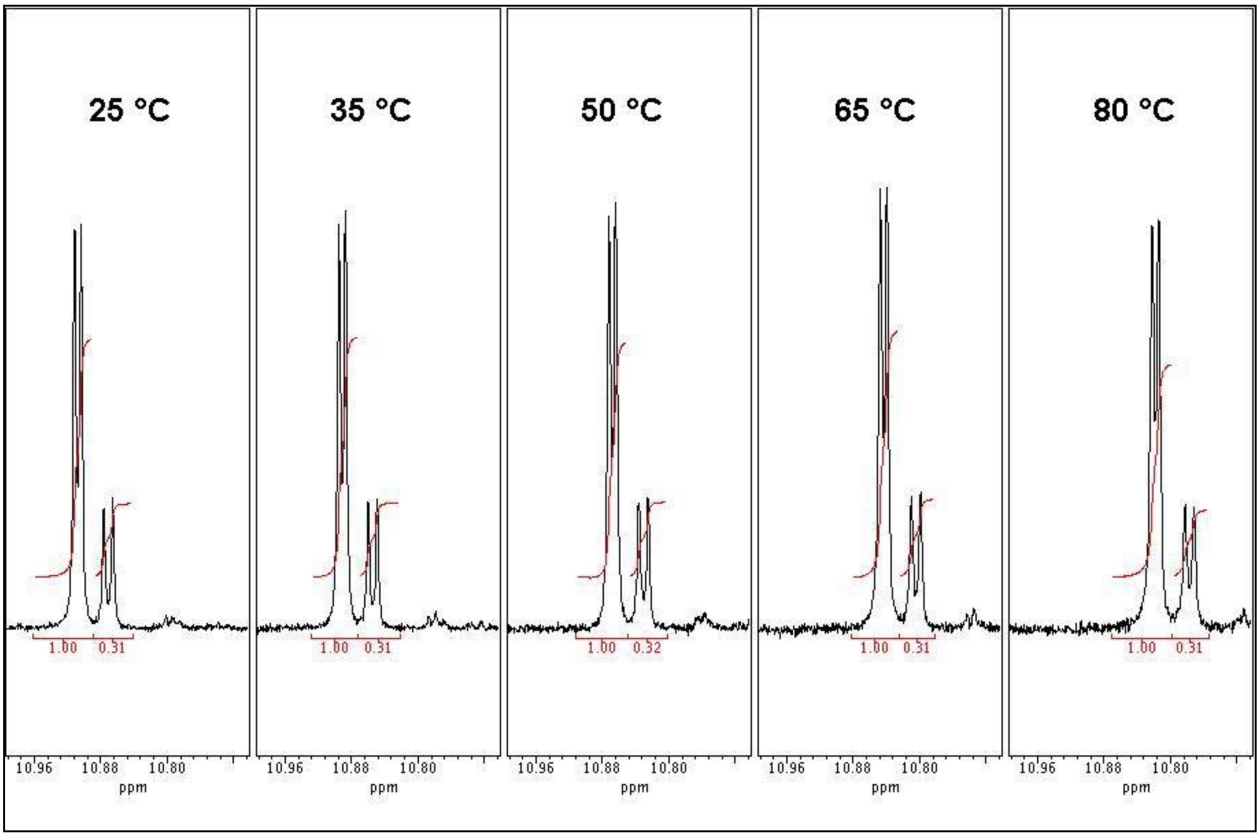

Figure S21: Expansion of NH region of ^{1}H spectra NMR of **U3G^2** (600MHz, DMSO-d$_6$) at different temperatures.

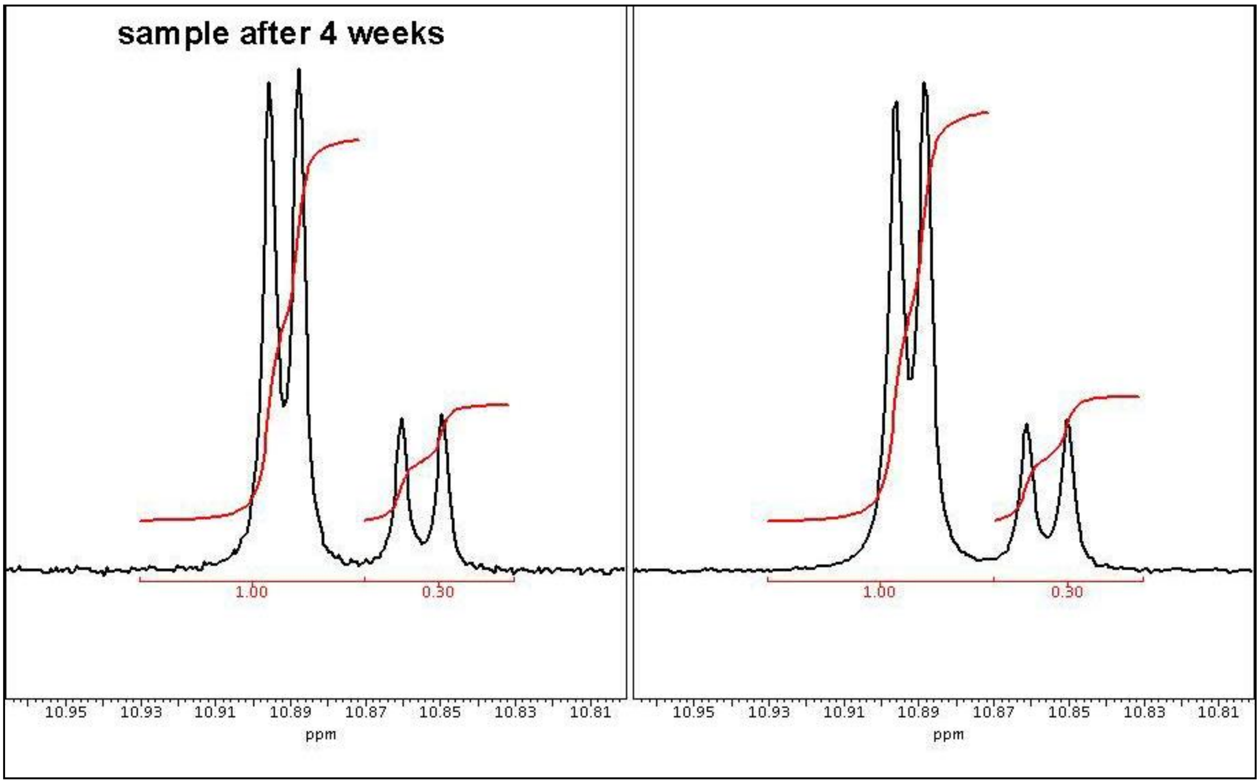

Figure S22: Expansion of NH region of ^{1}H spectra NMR of **U3G^2** after sample preparation and 4 weeks later (600MHz, DMSO-d$_6$).

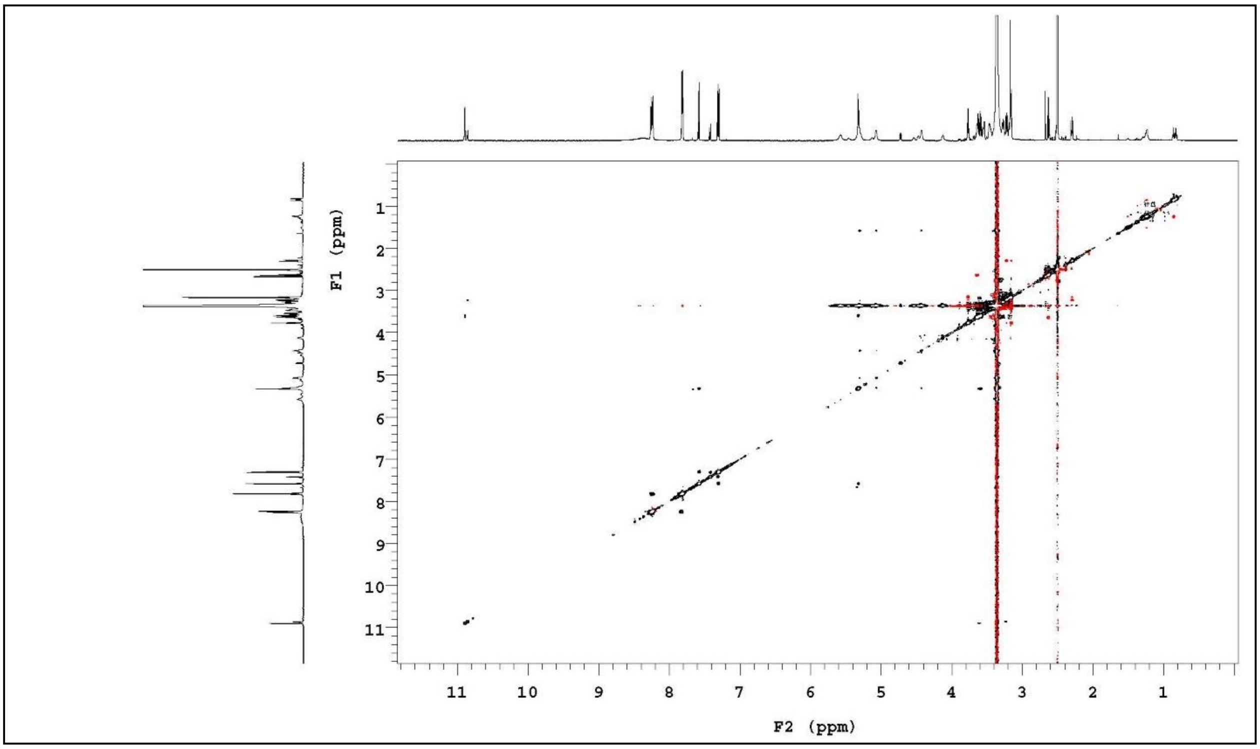

Figure S23: EXSY NMR of **U3G²** (600MHz, DMSO-d_6, 35 °C).
